# Endovenous ablation (radiofrequency and laser) and foam sclerotherapy versus conventional surgery for great saphenous vein varices

**DOI:** 10.1590/1516-3180.20141321T2

**Published:** 2014-02-01

**Authors:** Craig Nesbitt, Ron K. G. Eifell, Peter Coyne, Hassan Badri, Vish Bhattacharya, Gerard Stansby

## Abstract

**BACKGROUND:**

Minimally invasive techniques to treat great saphenous varicose veins include ultrasound-guided foam sclerotherapy (USGFS), radiofrequency ablation (RFA) and endovenous laser therapy (EVLT). Compared with conventional surgery (high ligation and stripping (HL/S)), proposed benefits include fewer complications, quicker return to work, improved quality of life (QoL) scores, reduced need for general anaesthesia and equivalent recurrence rates.

**OBJECTIVE:**

: To review available randomized controlled clinical trials (RCT) data comparing USGFS, RFA, EVLT to HL/S for the treatment of great saphenous varicose veins.

**METHODS:**

: *Search methods: *The Cochrane Peripheral Vascular Diseases (PVD) Group searched their Specialized Register (July 2010) and CENTRAL (The Cochrane Library 2010, Issue 3). In addition the authors performed a search of EMBASE (July 2010). Manufacturers of EVLT, RFA and sclerosant equipment were contacted for trial data. *Selection criteria: *All RCTs of EVLT, RFA, USGFS and HL/S were considered for inclusion. Primary outcomes were recurrent varicosities, recanalization, neovascularization, technical procedure failure or need for re-intervention, patient quality of life (QoL) scores and associated complications. Secondary outcomes were type of anaesthetic, procedure duration, hospital stay and cost. *Data collection and analysis: *CN, RE, VB, PC, HB and GS independently reviewed, assessed and selected trials which met the inclusion criteria. CN and RE extracted data. The Cochrane Collaboration's tool for assessing risk of bias was used. CN contacted trial authors to clarify details.

**MAIN RESULTS:**

Thirteen reports from five studies with a combined total of 450 patients were included. Rates of recanalization were higher following EVLT compared with HL/S, both early (within four months) (5/149 versus 0/100; odds ratio (OR) 3.83, 95% confidence interval (CI) 0.45 to 32.64) and late recanalization (after four months) (9/118 versus 1/80; OR 2.97; 95% CI 0.52 to 16.98), although these results were not statistically significant. Technical failure rates favoured EVLT over HL/S (1/149 versus 6/100; OR 0.12, 95% CI 0.02 to 0.75). Recurrence following RFA showed no difference when compared with surgery. Recanalization within four months was observed more frequently following RFA compared with HL/S although not statistically significant (4/105 versus 0/88; OR 7.86, 95% CI 0.41 to 151.28); after four months no difference was observed. Neovascularization was observed more frequently following HL/S compared with RFA, but again this was not statistically significant (3/42 versus 8/51; OR 0.39, 95% CI 0.09 to 1.63). Technical failure was observed less frequently following RFA compared with HL/S although this was not statistically significant (2/106 versus 7/96; OR 0.48, 95% CI 0.01 to 34.25). No randomised clinical trials comparing HL/S versus USGFS met our study inclusion criteria. QoL scores and operative complications were not amenable to meta-analysis.

**AUTHORS' CONCLUSIONS:**

Currently available clinical trial evidence suggests RFA and EVLT are at least as effective as surgery in the treatment of great saphenous varicose veins. There are insufficient data to comment on USGFS. Further randomized trials are needed. We should aim to report and analyze results in a congruent manner to facilitate future meta-analysis.

**This is the abstract of a Cochrane Review published in the Cochrane Database of Systematic Reviews (CDSR) 2011, issue 5, Art. No. CD005624. DOI:** 10.1002/14651858.CD005624.pub2 (http://cochrane.bvsalud.org/cochrane/main.php?lib=COC&searchExp=Endovenous%20and%20ablation%20and%20(radiofrequency%20and%20laser)%20and%20foam%20and%20sclerotherapy%20and%20versus%20and%20conventional%20and%20surgery%20and%20for%20and%20great%20and%20saphenous%20and%20vein%20and%20varices〈=pt).

**The full text is available from:** http://dx.doi.org/10.1002/14651858.CD005624.pub2.

**The abstract is also available in the Portuguese, French and Spanish languages from:** http://summaries.cochrane.org/pt/CD005624/ablacao-endovenosa-por-radiofrequencia-e-laser-e-escleroterapia-com-espuma-versus-cirurgia-convencional-para-o-tratamento-de-varizes.

## COMMENTS

With the advent of new techniques for treating varicose veins, many studies are needed in order to compare the new procedures with the gold-standard treatment, i.e. conventional surgery with removal of either the great or the small saphenous vein and excision of tributaries presenting insufficiency. In this review, many data were flawed or did not lead to a conclusion that would be capable of showing significant details regarding the best technique.

It can be expected that treatments with laser, radiofrequency or foam sclerotherapy may lead to recanalization of the treated veins, since these do not remove the veins but only stop the flow of blood through the lumen. Recurrence of varicose veins within four months suggests that there was an error in marking out the varicose veins before the operation and failure of the planned removal of the saphenous vein or the dilated tributaries. Some technical details of the surgery may differ, such as segmental removal of the great saphenous vein under general anesthesia. This procedure is not customary in many centers, and complete removal of the saphenous vein with intrathecal or regional blockade is preferred. Other extremely necessary data include comparison of the costs of the fiber laser and radiofrequency equipment, costs of procedures and costs of hospitalization when necessary.

**Marcelo Calil Burihan**. Professor of Vascular Surgery, Hospital Santa Marcelina, São Paulo; Professor of Anatomy, University of Santo Amaro, São Paulo; Professor of Anatomy, Santa Marcelina Medical School, São Paulo; Member of Sociedade Brasileira de Angiologia e de Cirurgia Vascular (SBACV), São Paulo, Brazil; and Member of the Society of Vascular Surgery (SVS), Latin American Chapter.
